# More of a Good Thing or Less of a Bad Thing: Gene Copy Number Variation in Polyploid Cells of the Placenta

**DOI:** 10.1371/journal.pgen.1004330

**Published:** 2014-05-01

**Authors:** James C. Cross

**Affiliations:** Department of Comparative Biology and Experimental Medicine, University of Calgary, Calgary, Alberta, Canada; University of Pennsylvania, United States of America

**Figure 1:**
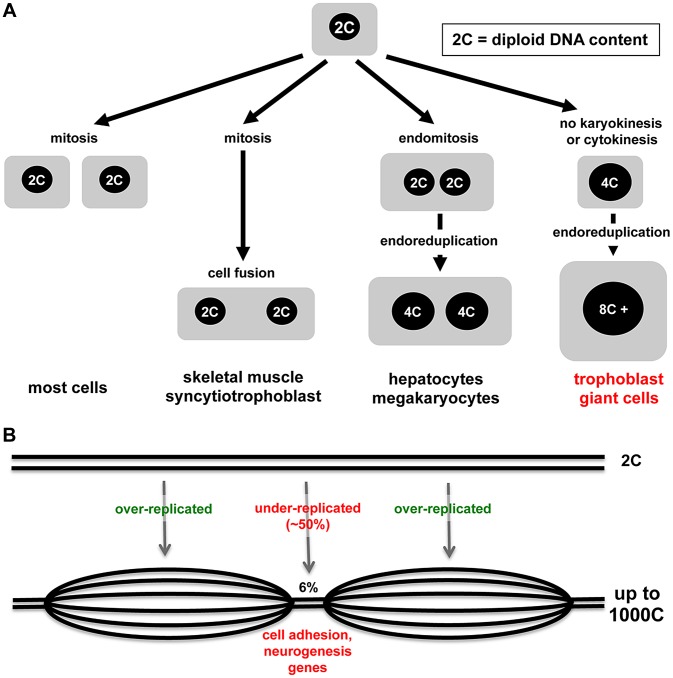
Modes of cell growth and polyploidy in mammals. (A) Diagram depicting different mechanisms underlying the formation of polyploid cells ranging from fusion of post-mitotic diploid cells, endomitosis (nuclear but not cell division), and endoreduplication. (B) Nuclei of polyploid trophoblast giant cells show selected small regions of under-replication (based on results from Hannibal et al.).

Nature demonstrates many interesting variations in cell cycles and cell growth (Figure 1A). Some tissues in animals, such as skeletal muscle and syncytiotrophoblast cells of the placenta, arise through the fusion of post-mitotic diploid progenitor cells to form multinucleated cells. Multinucleated cells such as liver cells can also arise through endomitosis, in which nuclei that have replicated DNA undergo division, but it is not followed by cytokinesis. Perhaps the most interesting example is endoreduplication, a cell cycle in which rounds of DNA synthesis are not coupled with intervening mitoses, usually resulting in cells with enlarged cytoplasm volume. Endocycles are a curiosity because completion of mitosis is required in mitotic cells before another round of DNA replication can occur. However, endoreduplication is observed widely in plants, protozoa, insects, and higher animals, and many different mitotic cell cycle alterations have been defined [1]. In mammals, the best-studied endoreduplicating cell type is the trophoblast giant cells (TGC) of the rodent placenta. In the accompanying paper, Hannibal and colleagues show that mouse TGCs don't endoreduplicate their genomes evenly and have developmentally regulated, under-replicated domains, suggesting that under-replication may be a mechanism to regulate cell function [2].

TGCs mediate uterine implantation of embryos, line the maternal blood space in the placenta, and secrete dozens of hormones thought to regulate maternal adaptations to pregnancy [3], [4]. Distinct TGC subtypes sit at different positions within the maternal blood space in the placenta [5], [6]. Parietal-TGCs, which form the interface with the maternal uterus, emerge first and achieve the highest ploidy [6]. After maternal circulation through the placenta is established, parietal-TGCs lie on the venous side as maternal blood leaves the placenta to enter uterine veins [5]. A distinct TGC subtype invades the maternal arteries that bring blood to the implantation site to replace the endothelial cells, while vascular spaces within the placenta itself are formed by morphogenesis of other TGC subtypes into tube-like structures [4].

The function of endoreduplication and polyploidy in TGCs remains a matter of debate, though it may be a way for the tissue to grow without the need to increase cell number [7], [8]—a matter of convenience at the maternal-fetal interface, which needs to develop rapidly. Several mouse mutants have defects in development and ploidy of TGCs [3], [8], but few of them cleanly distinguish the function of ploidy. Mutants in the E2F-7 and -8 cell cycle transcription factors show reduced TGC ploidy and cell size but, interestingly, have little change in TGC gene expression [9]–[11]. Functional models of TGC polyploidy are driven by the notion that over-replicated genes provide some advantage. In *Drosophila* polyploid cells, there are large regions of genome that are relatively over-replicated [1]. For many years it has been thought that TGCs endoreduplicate their entire genomes, but it would be hard to argue that the entire genome is important for TGC function. In vivo quantitation of TGC DNA content and of cells synchronously endoreduplicating in culture is consistent with a doubling of DNA content with each round [12]–[14]. In situ hybridization experiments using gene-specific probes suggest that TGC chromosomes are polytene due to the failure of replicated DNA strands to segregate [15]–[17]. In the 1990s, restriction landmark genomic scanning was developed to detect genome copy numbers and used to analyze CpG islands in rat placental TGCs [18]. At least 97% of the genome was similarly re-replicated, but the conclusion was limited by the technology of the day. In 2013, Sher et al. used array-based comparative genome hybridization to assess relative ploidies across the genome in TGCs dissected from mouse implantation sites and concluded that TGC genomes were uniformly duplicated [19]. This was also true for megakaryocytes and strikingly different than *Drosophila* polyploid cells [19].

With the advent of new technologies, it becomes possible to assess the genome with higher resolution and greater sensitivity, and so arrives the current paper from Julie Baker's lab [2]. Focusing on parietal-TGCs because of their high ploidy and using different technologies, Hannibal et al. demonstrate that some regions of the genome, though greater than diploid, are relatively under-replicated compared to the rest (Figure 1B) [2]. What accounts for the difference with the conclusions from Sher et al.? It may be that different TGC populations were sampled or that the approaches had different sensitivity. Comparative genome hybridization combined with whole genome sequencing showed that only 6% of the genome is under-replicated and, even in those regions, the under-replication is ∼50% compared to the rest of the genome [2]. Hannibal et al. show nicely that under-replicated regions are late replicating. Clearly the S-phase machinery must be kept away or inactivated in some regions. DNA synthesis in endoreduplicating TGCs is spread over ten to12 hours [12], [20], so there is ample opportunity to segregate regions of the genome.

The “if” and the “how” endoreduplication happens are only half of a good story; the “what” and the “why” are just as interesting. If under-replicated regions in TGCs were random, one could argue that under-replication was just a small error or a matter of convenience. What is intriguing about the data, however, is that the under-replication occurs in reproducible regions, detected both in TGCs in vivo and trophoblast progenitors differentiated in culture. Analysis of the 47 under-replicated regions shows that they are enriched for some classes of genes, including those involved in cell adhesion and development of the nervous system. Trophoblast progenitor cells reduce cell-cell adhesion as parietal-TGCs develop [21]–[23]. Microarray data from cultured mouse trophoblast stem cells (http://www.ncbi.nlm.nih.gov/sites/GDSbrowser?acc=GDS3948) show expression of “nervous system genes” like the nerve guidance protein Slit. Human trophoblast cells also express Slit and it is overexpressed in the placenta in preeclampsia [24], a pregnancy complication associated with defects in trophoblast cell function. This new evidence might start to change the thinking about endoreduplication. Instead of thinking that more copies of genes that are good for TGC function is the goal, perhaps it is important to reduce duplication of genes that impair TGC function. Let the experiments begin.
